# Supplementary material for: Evaluation of Small Molecule Combinations against Respiratory Syncytial Virus In Vitro
Source: Molecules. 2021 Apr 29;26(9):2607. doi: 10.3390/molecules26092607 (PMC8125180; doi:10.3390/molecules26092607)
Supplement: Supplementary file 1 [file molecules-26-02607-s001.zip › molecules-1098374-supplementary.pdf]

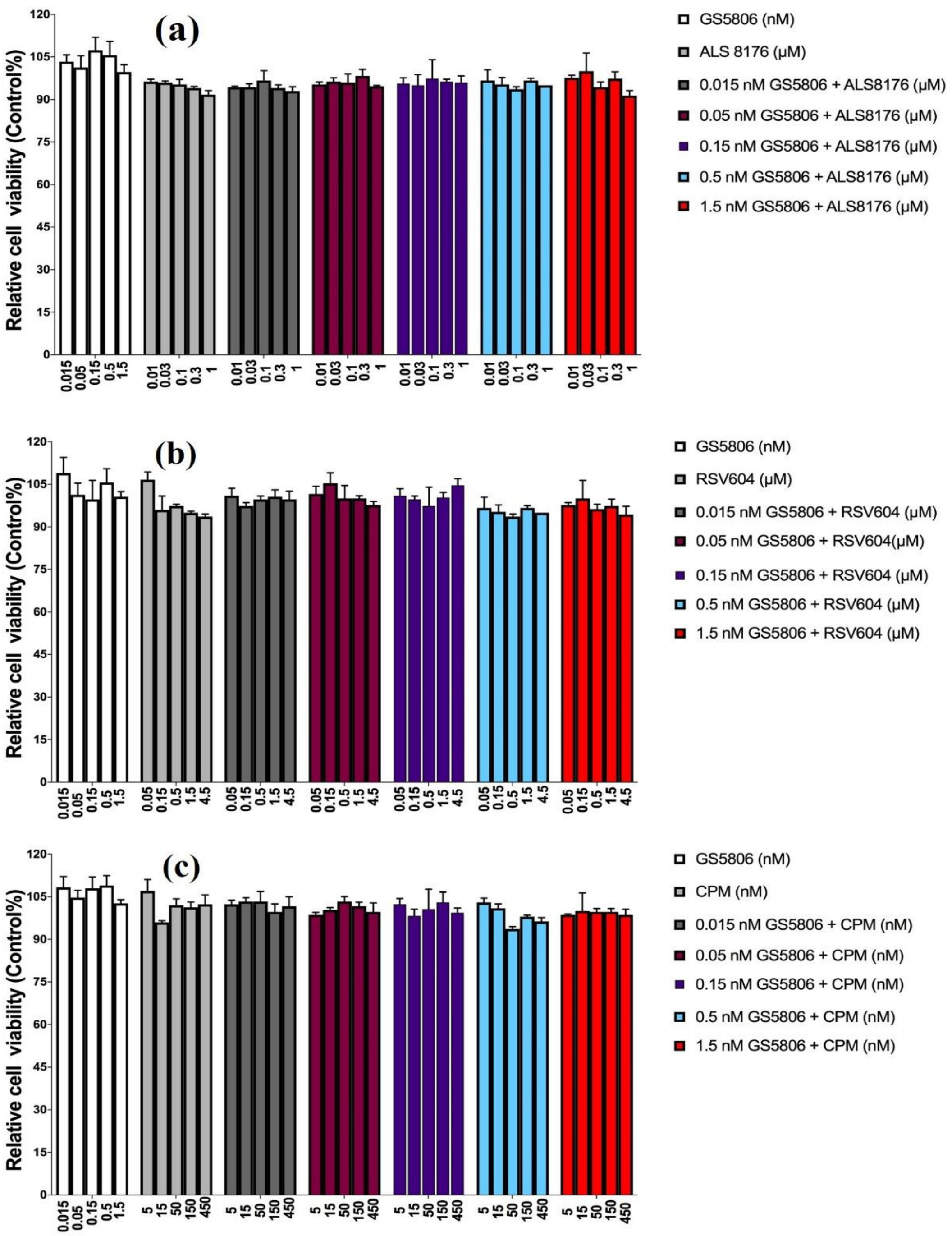

Figure S1. Cell viability assays of GS5806–RdRp inhibitor combinations. (a) GS5806–ALS8176, (b) GS5806–RSV604 and (c) GS5806–CPM. The results are representative of three independent experiments performed in triplicate (mean ± s.e.m.).

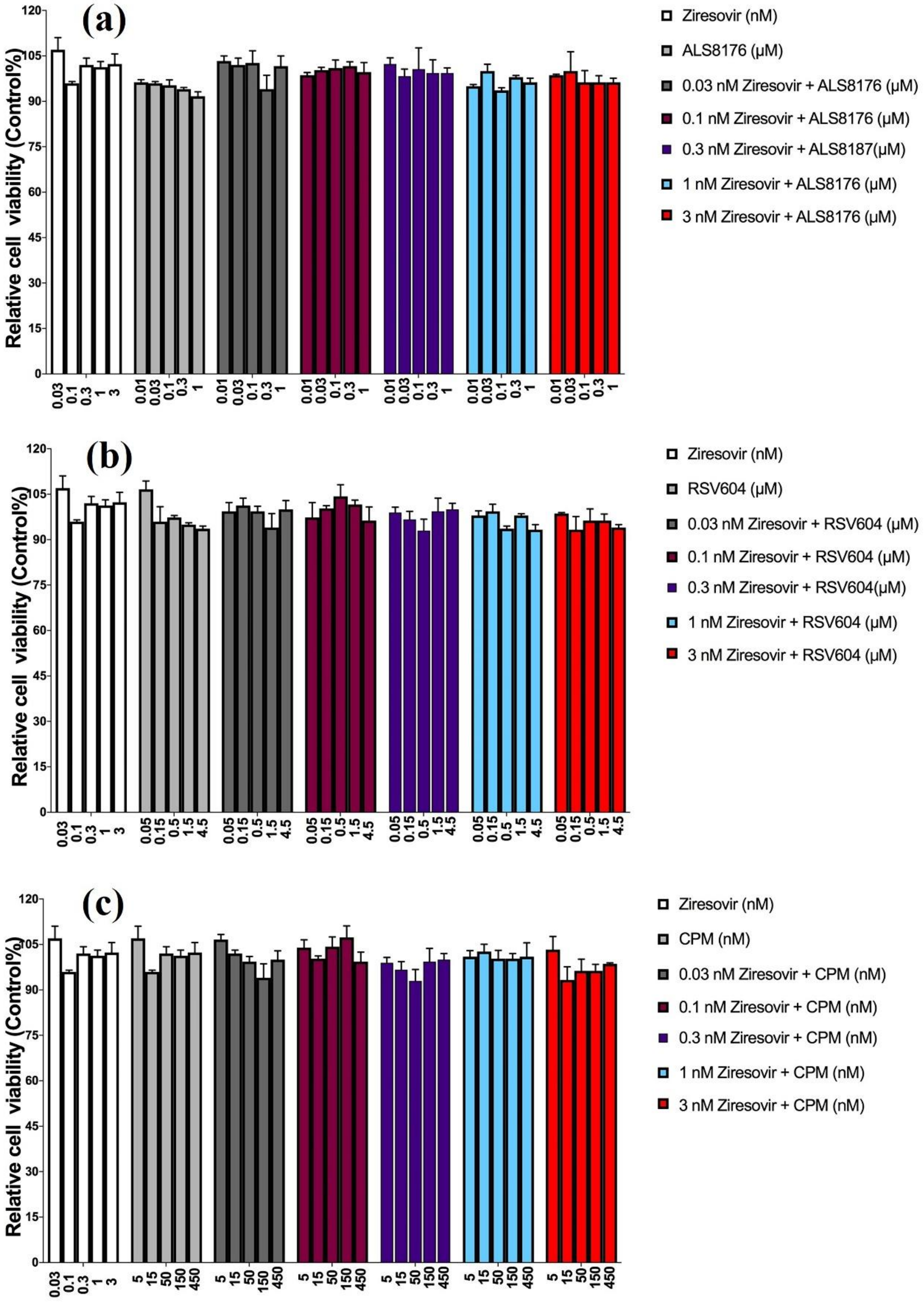

Figure S2. Cell viability assays of Ziresovir–RdRp inhibitor combinations. (a) Ziresovir–ALS8176, (b) Ziresovir–RSV604 and (c) Ziresovir–CPM. The results are representative of three independent experiments performed in triplicate (mean ± s.e.m.).

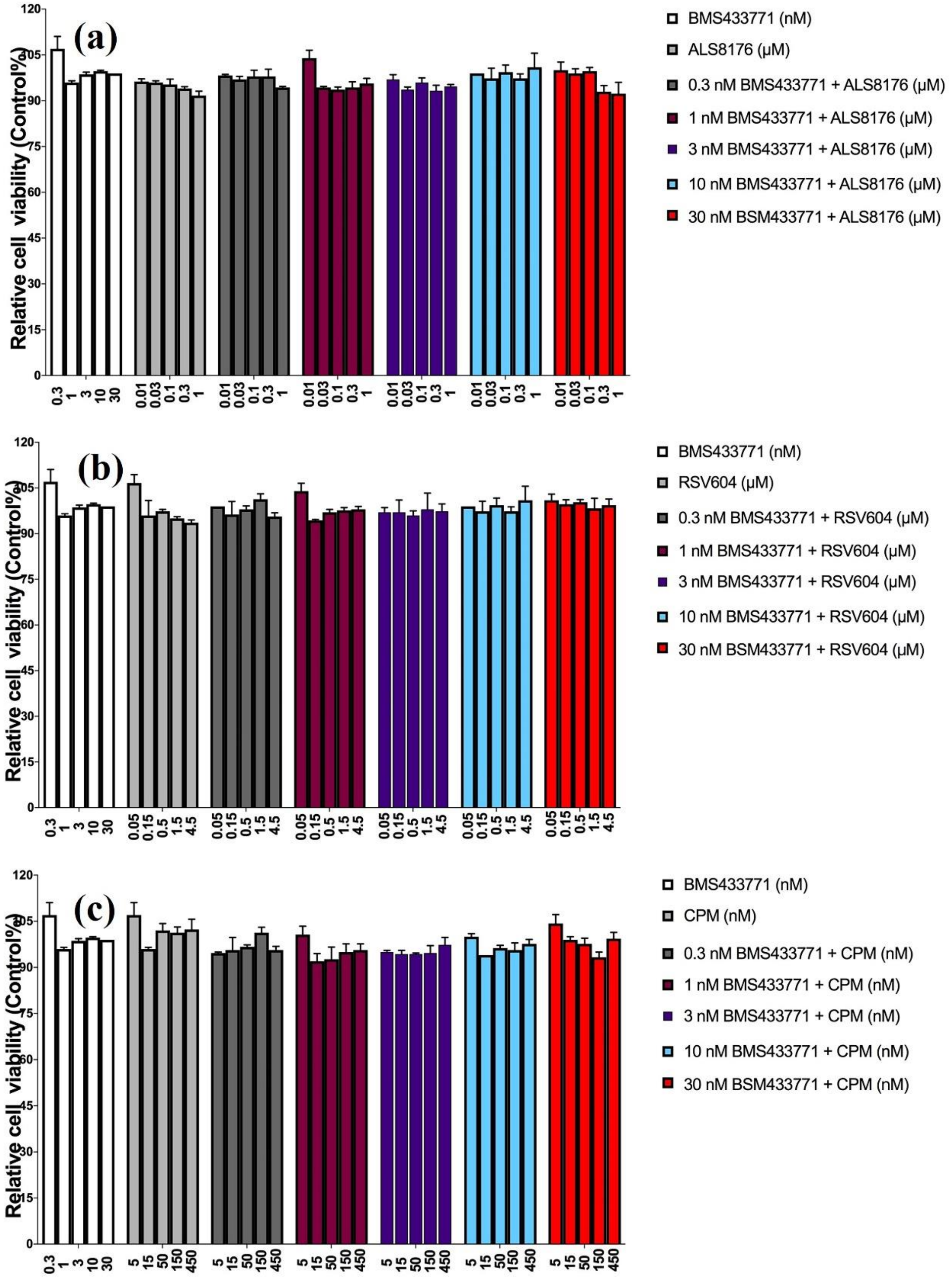

Figure S3. Cell viability assays of BMS433771–RdRp inhibitor combinations. (a) BMS433771–ALS8176, (b) BMS433771–RSV604 and (c) BMS433771–CPM. The results are representative of three independent experiments performed in triplicate (mean ± s.e.m.).

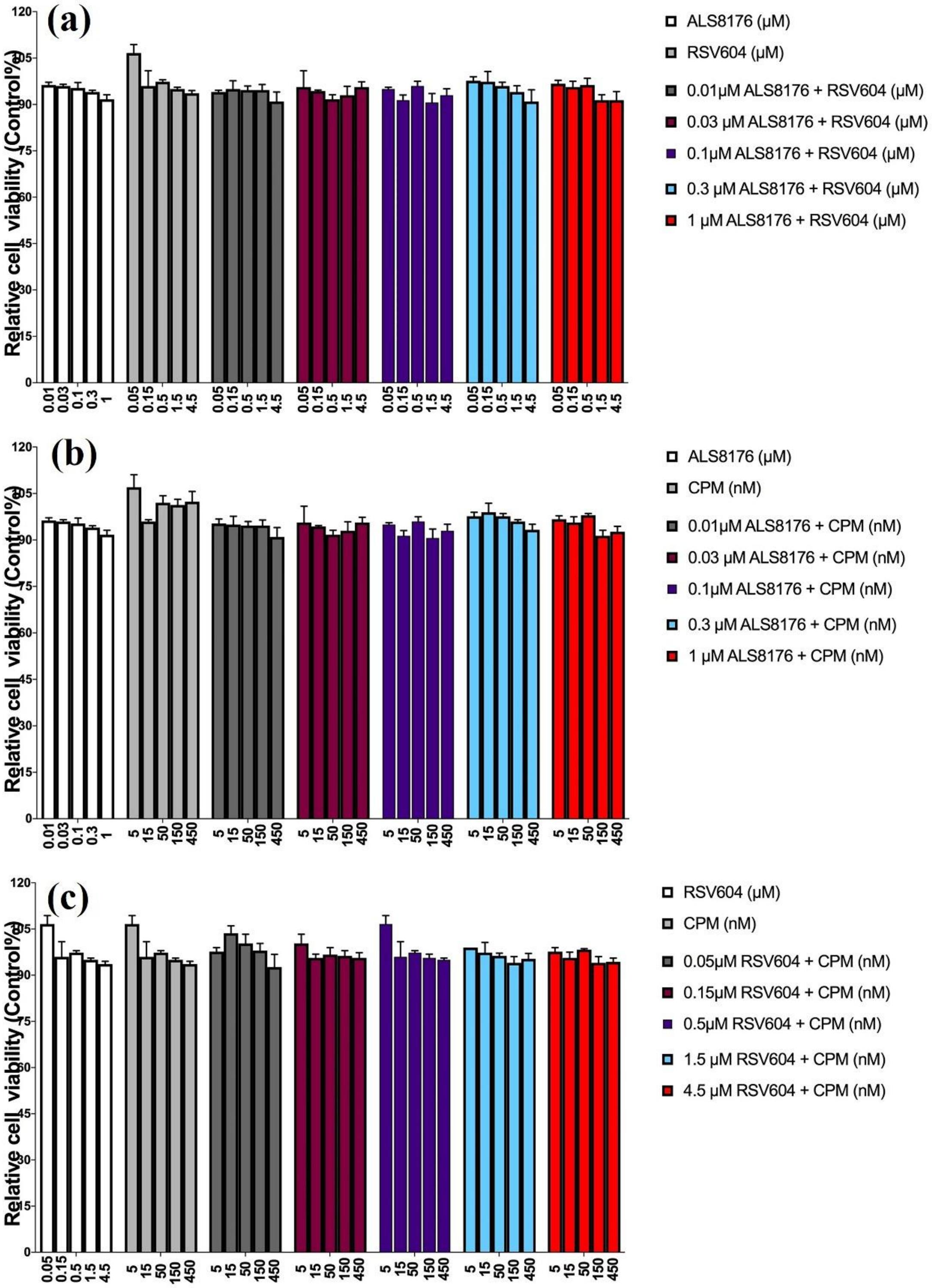

Figure S4. Cell viability assays of combinations between RdRp inhibitors. (a) ALS8176–RSV604, (b) ALS8176–CPM and (c) RSV604–CPM. The results are representative of three independent experiments performed in triplicate (mean ± s.e.m.).

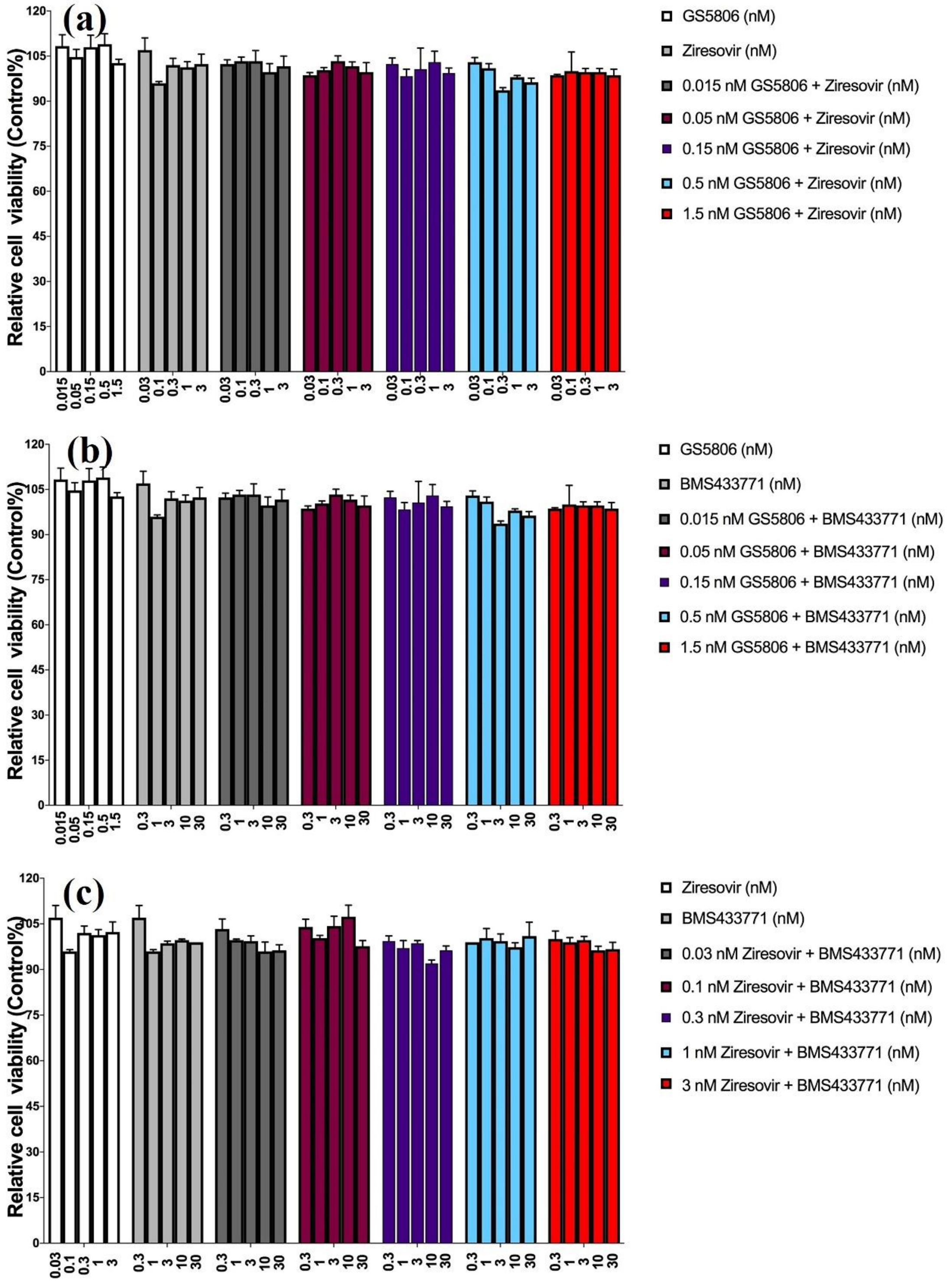

Figure S5. Cell viability assays of combinations between fusion inhibitors. (a) GS5806–Ziresovir, (b) GS5806–BMS433771 and (c) Ziresovir–BMS433771. The results are representative of three independent experiments performed in triplicate (mean ± s.e.m.).
